# Retraction: Foliar application of mepiquat chloride and nitrogen improves yield and fiber quality traits of cotton (*Gossypium hirsutum* L.)

**DOI:** 10.1371/journal.pone.0273466

**Published:** 2022-08-31

**Authors:** The PLOS ONE Editors

The *PLOS ONE* Editors retract this article [1] because it was identified as one of a series of submissions for which we have concerns about authorship, competing interests, and peer review. We regret that the issues were not addressed prior to the article’s publication.

HA, MAW, AS, SAT, SI, MSE, and MC did not agree with the retraction. YL responded but expressed neither agreement nor disagreement with the editorial decision. MFS and JA either did not respond directly or could not be reached.
